# Experience delivering an integrated service model to people with criminal justice system involvement and housing insecurity

**DOI:** 10.1186/s12889-023-15108-w

**Published:** 2023-02-02

**Authors:** Olivia Baker, Chevaughn Wellington, Carolina R. Price, DeShana Tracey, Lindsay Powell, Sara Loffredo, Silvia Moscariello, Jaimie P. Meyer

**Affiliations:** 1 Yale School of Medicine, Section of Infectious Diseases, New Haven, CT USA; 2 Boston Medical Center, Boston, MA USA; 3 Yale School of Nursing, New Haven, CT USA; 4 Liberty Community Services, Inc, New Haven, CT USA; 5 Yale School of Public Health, Chronic Disease Epidemiology, New Haven, CT USA

**Keywords:** Housing, Criminal justice, Integrated service delivery

## Abstract

**Background:**

People returning to communities from prison or jail face stressors related to securing housing, including discrimination, restrictions based on prior felony convictions, and limited economic and social resources. Existing housing programs can effectively reduce housing instability but often do not fully address the needs of people involved in the criminal justice system experiencing homelessness who often have co-occurring chronic medical issues, and psychiatric and substance use disorders.

**Methods:**

Project CHANGE is an ongoing program to deliver person-centered, integrated care and services to individuals involved with the criminal justice system and experiencing homelessness. Applying a Screening, Brief Intervention, (Referral to) Treatment framework, a comprehensive needs assessment is followed by delivery of intensive housing and vocational case management; and psychiatric, substance use, and medical services in a single location by an interdisciplinary team. Participants are followed with study interviews for 12 months. The current analysis was designed to assess the baseline characteristics and needs of the sample population, and the intensity of contact required for integrated service delivery.

**Results:**

Between November 2019 and September 2021, 86 participants were enrolled, of whom 64% had been released from prison/jail in the past 6 months; the remainder were on parole, probation, or intensive pretrial supervision. Participants were unstably housed (64%) or residing outdoors (26.7%) or in a shelter (24.4%). Most participants had high medical need and frequent healthcare engagement through outpatient and emergency department visits. Most participants were at-risk for clinical depression, and half were diagnosed with anxiety, dissociative, stress-related, somatoform, and other non-psychotic psychiatric disorders. Over 12-month follow-up, the interdisciplinary team made over 500 contact encounters, over half of which resulted in direct services provided, including obtaining vital documents for homelessness verification, housing applications, and employment coaching.

**Conclusion:**

Navigation of services can be particularly challenging for individuals experiencing criminal justice involvement, homelessness, and co-occurring medical, psychiatric, and substance use issues, which can be addressed holistically in an integrated service model. Integrated service delivery was time-, resource-, and staffing-intensive, and challenged by the COVID-19 pandemic, requiring innovative solutions to sustain participant engagement.

**Supplementary Information:**

The online version contains supplementary material available at 10.1186/s12889-023-15108-w.

## Background

Incarceration has been called a “revolving door” of homelessness. People returning to communities from prison or jail often face barriers to housing that include discrimination, restrictions on public housing based on prior felony convictions, co-occurring psychiatric and substance disorders, lack of social support, and lack of income [1–3]. Stressors related to securing employment, healthcare, and social support pose additional challenges to people involved in the criminal justice system (CJS) [1, 2, 4–6]. In turn, people who are homeless are often criminalized and charged with minor offenses that return them to closed detention settings.

Opportunities for successful community re-entry are reduced further when people return to high-crime, impoverished, and under-resourced neighborhoods [7]. Social support is vital to successful community re-entry in terms of assisting with housing, transportation, and job networking, but some formerly incarcerated individuals may be isolated from systems of social support because of conditions of parole, interpersonal conflict, or lack of social connections [1, 8]. As a result, they may remain socially isolated and essentially left to reside in the streets, shelters, or transitional housing settings [3, 5, 8]. In addition to other comorbidities, people involved in the CJS disproportionately experience HIV and HIV risk [9, 10]. For people living with HIV and co-occurring psychiatric and substance use disorders, community reentry can be chaotic and threaten continuity of HIV care, which has important individual and public health impact [11–13]. For others, this reentry period is also associated with increased HIV risk behaviors, including relapse to substance use, and increased mortality primarily attributable to opioid overdose [14]. The focus of most community-based programs for formerly incarcerated individuals is to reduce recidivism (that is, reduce re-arrest, reconvictions, or return to incarceration), address homelessness, or increase uptake of treatment services [6, 15–17]. Yet services for community reintegration are often fragmented, posing challenges to navigation [18, 19].

Community-based housing interventions that address acute or chronic homelessness should be tailored for individuals with co-occurring psychiatric and substance use disorders, including people involved in the CJS. Overall, there are few housing interventions specific to people involved in the CJS that have been rigorously evaluated, and numerous missed opportunities to prevent homelessness as part of discharge planning from prison or jail [15, 20].

To address the need for comprehensive integrated services in this population, we developed Project CHANGE (Comprehensive Housing and Addiction Treatment Network of Greater New Haven), a SAMHSA-funded service project to expand and enhance the local delivery of integrated housing, behavioral health, and addiction treatment services for highly vulnerable populations involved in the CJS. Project CHANGE is based on the premise that holistically addressing the complex needs of this population will reduce recidivism, improve physical and mental health outcomes, promote economic security, improve social support, and enhance overall quality of life. Here, we describe how Project CHANGE delivers coordinated services and present findings from a baseline comprehensive needs assessment, and describe intensity and type of service delivery over the subsequent 12 months. We also describe challenges to integrated service delivery presented by the high demand on resources and by the COVID-19 pandemic.

## Methods

### Aim and design

**Fig. 1:**
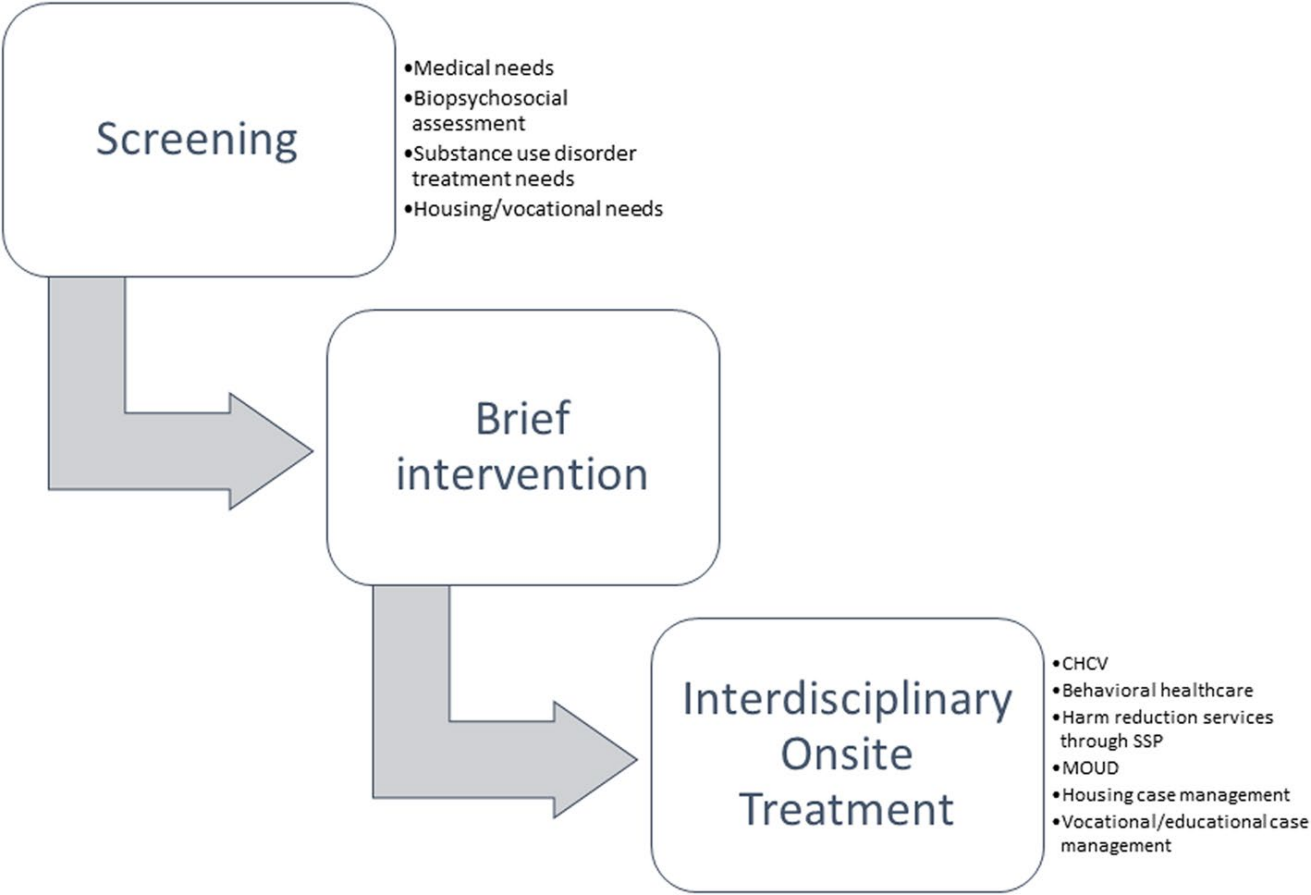
Screening, brief intervention, and (referral to) treatment in project CHANGE for integrated service delivery Legend: *CHCV* community healthcare van (mobile medical clinic); *MOUD* medications for opioid use disorder; *SSP* syringe services program

The overall aim of Project CHANGE is to integrate housing and health services for people who are involved in the CJS. Services are delivered in an integrated framework to promote a person-centered “one-stop shop,” in which individuals have access to behavioral healthcare, case management, and medical services in one location (Fig. 1.) Enrollment and data collection are ongoing. The Behavioral Model for Vulnerable Populations underpins the intervention and supposes that healthcare utilization and health outcomes are driven by predisposing, enabling, and need factors that are modifiable (Supplemental Figure) [21].

### Study setting

Project CHANGE serves individuals in the Greater New Haven area of Connecticut who are experiencing co-occurring homelessness, psychiatric, and substance use disorders. Homelessness and housing instability remain a pervasive problem in the city of New Haven, an urban center with high rates of poverty and the second highest drug overdose death rate in the state [22, 23]. Housing, drug treatment, and mental health services are traditionally siloed and demands exceed availability. As a result, untreated comorbid medical, psychiatric, and substance use disorders (SUD) converge with homelessness and other socioeconomic disparities including CJS-involvement, resulting in poor outcomes for individual and public health.

Project CHANGE is based out of a university-owned site that houses the New Haven Syringe Services Program, a behavioral health services team, and multiple ongoing clinical research programs related to HIV prevention and treatment, and treatment of SUD, for people involved in the CJS. The site is also home to the Community Healthcare Van—a mobile medical clinic that travels throughout the city to deliver onsite primary care and treatment for substance use disorders, HIV, and Hepatitis C [24]. This project added to the array of available services by partnering with Liberty Community Services (hereafter referred to as “Liberty”), a program designed to end homelessness in the Greater New Haven area, including among individuals living with HIV, psychiatric, and substance use disorders [25]. Liberty offers housing case management services, eviction prevention, security deposits, and employment services.

### Participant recruitment and enrollment

Participants are primarily recruited through community outreach at the local library, COVID hotels (where individuals were placed during the COVID-19 pandemic when shelters were closed), health fairs, probation offices, soup kitchens, and food pantries. Research staff are onsite intermittently at these various community locations for recruitment and to post promotional material. Promotional materials are also posted at our research offices (co-located with the syringe service program and Community Healthcare Van) and provided by discharge staff at local prisons and jails to individuals who are close to date of planned release and plan to return to New Haven. Interested participants can call a secure study phone line to complete eligibility screening (or call upon release for those currently incarcerated) or can complete eligibility screening in person with CHANGE staff onsite in the community.

Participants are eligible to take part in the study if they are: 1) residing or planning to reside in New Haven; 2) at least 18 years old; 3) involved in the CJS (“justice-involved”); 4) homeless or unstably housed; 5) experiencing co-occurring substance use or psychiatric disorders; and 6) able to provide informed consent. Participants are excluded if they have a legal conservator of person, are unwilling or are not able to independently complete the informed consent process or have previously been excluded from Liberty based on safety concerns by staff. Justice-involvement is defined as having been released from prison or jail within 6 months or on community supervision (probation, parole, intensive pretrial supervision) and is verified through the state’s court and judicial online database that is publicly available. To be more inclusive, we additionally include people who are not justice-involved but who are at-risk for justice-involvement and HIV, by virtue of having traded sex for drugs, money, food, or shelter or having injected drugs in the past 6 months (though, to date, all enrolled participants have met justice-involvement criteria). We apply the Housing and Urban Development (HUD) definitions of homelessness, including literally homeless (Category 1: residing in a place not meant for human habitation or in a shelter); at imminent risk of homelessness (Category 2: in an unstable housing arrangement, such as couch-surfing, doubled-up, in transitional housing, or owing significant back-rent); and fleeing/attempting to flee domestic violence (Category 4) [26]. People who are interested and eligible for enrollment complete written informed consent and sign release of information forms for Liberty and other community health and service agencies. Potential participants who are referred while incarcerated complete eligibility screening and enrollment procedures only after returning to the community.

### Measures

After completing enrollment procedures, integrated services are initiated using an evidence-based intervention known as Screening Brief Intervention Referral to Treatment (or Treat) (SBIRT), as shown in Fig. 1 [27]. Screening is done in a single interview with a research assistant in English or Spanish who enter data into REDCap electronic data capture tool, hosted at Yale University [28, 29]. The interview is held in person or virtually during COVID-19 restrictions. Baseline study assessments cover the following domains:

#### Demographics

Demographics are collected using The Government Performance and Results Act (GPRA), a standardized reporting platform for SAMHSA grantees. Section A identifies participants’ age, gender, sex assigned at birth, and race/ethnicity. Section D identifies education, employment, and income. Section C indicates participants' family and living situation.

#### Criminal justice involvement

The Addiction Severity Index (ASI) is used at baseline to assess and address the severity of an individual’s substance use in 7 domains: medical status, employment and support, drug use, alcohol use, legal status, family/social status, and psychiatric status [30]. We use the Addiction Severity Index-Legal component (ASI-L) to assess participants' current and past criminal justice involvement, based on its predictability for future incarceration and our experience using it to assess criminal justice involvement history in prior clinical trials [31, 32]. Participants identify their current parole or probation status.

#### Care for medical issues

Section F of the GPRA is a self-report survey of reasons for and number of inpatient, outpatient, and emergency department visits over the past 30 days, and HIV testing status. We do not collect data on testing location or reasons for testing. Participants also self-report Hepatitis C testing status as a part of a medical screen. The 12-item Short Form Survey (SF-12) assesses health-related quality of life through group comparisons involving multiple health dimensions [33]. Scores range between 0–100, where higher scores suggest better health-related quality of life [34, 35].

#### Behavioral health care

At baseline, depression severity is assessed with the 9-item Patient Health Questionnaire (PHQ-9), in which participants indicate whether problems over the past two weeks occur “not at all,” “several days,” “more than half the days,” or “nearly every day.” Responses are summed for an overall score; a score ≥ 20 indicates severe depression [36]. We also assess depressive symptoms using the Center for Epidemiologic Studies Depression Scale (CES-D), a 20-item self-report measure divided into subscales: sadness (dysphoria), loss of interest (anhedonia), appetite, sleep, thinking/concentration, guilt (worthiness), tired (fatigue), movement (agitation), and suicidal ideation [37]. Responses are summed for an overall score; a score ≥ 16 indicates risk for clinical depression [37]. Participants meet with an onsite clinical social worker for a complete biopsychosocial assessment, that includes a clinical interview and identification of primary, secondary, and/or tertiary behavioral health diagnoses as defined by the Diagnostic and Statistical Manual of Mental Disorders, Fifth Edition (DSM-5).

#### Substance use

The National Institute of Drug Abuse- Modified Alcohol, Smoking, and Substance Involvement Screening Test (ASSIST) is used to assess substance use severity. Participants identify the types of substances they have used in their lifetime, past year, and past three months; a score > 27 indicates a high substance use risk level [38]. The Drug Abuse Screening Test (DAST-10) assesses substance use in the past year (excluding tobacco and alcohol) [39]. A DAST-10 score > 9 indicates severe substance use and a need for intensive intervention [39, 40].

### Intervention: service integration

Needs assessment is followed by integrated service delivery. Based on the needs identified through the Screening component of SBIRT, people are connected to services onsite as much as possible, and offsite if needed or participant preferred (Fig. 1). Participants who report unmet medical or behavioral health needs are given a warm handoff (in which two healthcare or service providers communicate about the patient in front of the patient) to the mobile medical clinic and onsite behavioral health providers; for harm reduction needs, services are delivered through our onsite SSP; and for housing and vocational needs, services are delivered through our collaborating housing service provider. Participants who report active involvement with an outside agency providing these same services are not referred internally and are instead asked to sign a Release of Information form to help coordinate services.

Each client is assigned a Liberty Intensive Housing Case Manager and a Vocational Case Manager. Liberty’s services are usually delivered at a separate location, but for this project, Liberty case managers are onsite (virtually or in person) to meet with participants and conduct a standardized housing assessment that is used by Liberty to identify agency and state programs to address individual’s needs (such as rapid rehousing, eviction prevention, and employment services). Liberty homelessness verification data are not used to include or exclude participants in Project CHANGE as they are already enrolled once Liberty homelessness verification occurs. The Intensive Case Manager meets with enrolled participants regularly to assist in the crisis stabilization process, including contacting 211 to obtain shelter, and providing homeless verification letters, and any necessary referrals. Employment/Educational Vocational Case Managers facilitate access to resources for employment, training, and education to increase their earning potential and they maintain a clearinghouse of information on community resources. They also offer vocational and educational programming. Participants work with the case managers for a minimum of 12 months, and each participant can have up to 2 case managers at any time, working on different aspects of service coordination, depending on their identified needs.

Encounter forms in REDCap track all services provided to participants and are reviewed regularly by the study team for quality assurance, the major metric for which is completion. The interdisciplinary clinical, research, housing/vocational service, and behavioral health team meet monthly via Zoom to ensure fidelity to the study protocol, discuss milestones and encounters with participants, identify unmet needs, and coordinate care.

Participants are followed for 12 months, and research study interviews occur at baseline, month 6, and month 12. Participants are compensated $20 for each completed study interview. The study team conducts brief check-ins with participants by phone or text at months 3 and 9 to promote client engagement and retention and update their file with a brief description of their current housing, employment, medical, and mental health status. More frequent communication may be warranted depending on the client’s specific needs. Additionally, case management and further referrals may also be made if needed. The current analysis used baseline data for the first 86 participants enrolled. Further longitudinal analysis on implementation outcomes will be reported once data collection is complete.

### Analysis

We performed a descriptive analysis of baseline data from all enrolled participants to characterize the sample in terms of demographic factors, criminal justice involvement, and medical, psychiatric, and substance use treatment needs and engagement. We then calculated the intensity and type of service delivery in terms of encounters with Project CHANGE staff over the 12 months of follow-up. All analyses were performed using IBM SPSS Statistics, version 28.0 Armonk, NY: IBM Corp.

## Results

### Baseline characteristics and needs

As shown in Table 1, between November 2019 and September 2021, 86 participants were newly enrolled in Project CHANGE. The mean age of the cohort was 43.81 years old (SD 9.82). Overall, 48.8% of participants identified as cis-female, 48.8% as cis-male, and 2.3% as transgender (1 trans-male and 1 trans-female). The sample was racially/ethnically diverse– participants identified as white (59.3%), Black (40.7%), Hispanic/Latinx (19.8%), and American Indian (9.3%). Participants’ education status was evenly distributed with 29.2% receiving less than a high school education, 38.4% receiving a high school diploma/equivalent, and 32.6% receiving more than high school education. At baseline, most participants were unemployed (65.1%), and half were unstably housed (e.g., halfway house, residential housing, etc.), with the remainder meeting criteria for literal homelessness.

**Table 1:** Baseline characteristics of program participants (*N* = 86)

Characteristic	Mean (SD) or N (%)
**Mean Age (SD)**	43.81 (9.82)
**Sex and gender**	
Cis-male	42 (48.8)
Cis-female	42 (48.8)
Trans-male	1 (1.2)
Trans-female	1 (1.2)
^a^ **Race/Ethnicity**	
Black	35 (40.7)
White	51 (59.3)
American Indian	8 (9.3)
Hispanic/Latino	17 (19.8)
**Education status**	
Less than High School	25 (29.2)
High School Diploma/Equivalent	33 (38.4)
More than High School	28 (32.6)
**School/Job training program**	
Not enrolled	75 (87.2)
Enrolled, Full Time	2 (2.3)
Enrolled, Part Time	9 (10.5)
**Employment status**	
Employed Full-time (35 + Hours per week)	7 (8.1)
Employed Part-time	11 (12.8)
Unemployed, looking for work	35 (40.7)
Unemployed, disabled	16 (18.6)
Unemployed, not looking for work	5 (5.8)
**Housing status**	
Street/Outdoors	23 (26.7)
Shelter	21 (24.4)
Unstably Housed	42 (48.8)
**Current criminal justice involvement**	
Parole	10 (11.6)
Probation or Intensive Pretrial Supervision	42 (48.8)
Released from Prison/Jail Within 6 Months	55 (64.0)
**Past 30-day inpatient treatment**	
Physical/medical issues	4 (4.7)
Mental health issues	3 (3.5)
Alcohol or Substance Use	7 (8.1)
**Past 30-day outpatient treatment**	
Physical/medical issues	21 (24.4)
Mental health issues	27 (31.4)
Alcohol or Substance Use	42 (48.8)
**Past 30-day emergency department treatment**	
Physical/medical issues	18 (20.9)
Mental health issues	5 (5.8)
Alcohol or Substance Use	4 (4.7)
**Last tested for HIV**	
< 6 months	43 (50.0)
> 6 months	37 (43.0)
Don’t Know/Unsure/Missing	6 (7.0)
**Last tested for Hepatitis C**	
≤ 1 Year	58 (67.4)
≥ 1 Year	24 (27.9)
Don’t Know/Unsure	4 (4.7)
**Mean health-related quality of life (SD) **	
Physical Health	42.23 (11.98)
Mental Health	37.85 (12.04)
**Depression severity**	
Mean Score (SD)	10.86 (7.67)
Severe Depression (%)	17 (19.8)
**Risk for clinical depression**	
Mean Score (SD)	26.12 (13.65)
At-risk	62 (72.1)
**Behavioral health diagnosis**	
Alcohol Related Disorders	25 (29.1)
Opiate Related Disorders	27 (31.4)
Cocaine Related Disorders	25 (29.0)
Bipolar Disorder	14 (16.3)
Major Depressive Disorder	9 (10.5)
Anxiety, Dissociative, Stress-Related, Somatoform, and other Non-Psychotic Mental Disorder	39 (45.3)
**Past-year substance use**	
Cannabis	85 (98.8)
Cocaine	74 (86.0)
Prescription Stimulants	22 (25.6)
Methamphetamine	17 (19.8)
Inhalants	17 (19.8)
Sedatives or Sleeping Pills	38 (44.2)
Hallucinogens	43 (50.0)
Street Opioids	50 (58.1)
Prescription Opioids	49 (57.0)
**Past 3-month substance use risk level**	
Low	7 (8.1)
Moderate	35 (40.7)
High	43 (50.6)
Missing	1 (1.2)
**Past-year substance use severity**	
No problems	17 (19.8)
Low Level	8 (9.3)
Moderate Level	9 (10.5)
Substantial Level	50 (58.1)
Missing	2 (2.3)

^a^ Participants can select more than one

At screening, 64.0% of participants had been released from prison/jail in the past 6 months and 48.8% were on probation, parole, or intensive pretrial supervision. Participants regularly engaged with healthcare settings, including through inpatient, outpatient, and emergency department settings, with high rates of recent HIV and HCV testing. Medical needs were high as evidenced by suboptimal physical and mental health-related quality of life that was significantly below mean scores for the U.S. general population. Psychiatric disorders were common, and anxiety, dissociative, stress-related, somatoform, and other non-psychotic disorders were the most prevalent behavioral health diagnoses. Cannabis and cocaine were the most used substances in the past year. Overall, participants experienced a high substance use risk level in the past 3-months and past-year.

### Intensity and type of service delivery

All enrolled participants were offered case management, medical and behavioral health services to meet identified needs. Approximately 97% of participants were given a warm handoff to Liberty staff for employment or vocational case management, 85% to the behavioral health team for a biopsychosocial needs assessment, and 82% to the Community Healthcare Van for medical tests, treatment, or services.

**Fig. 2:**
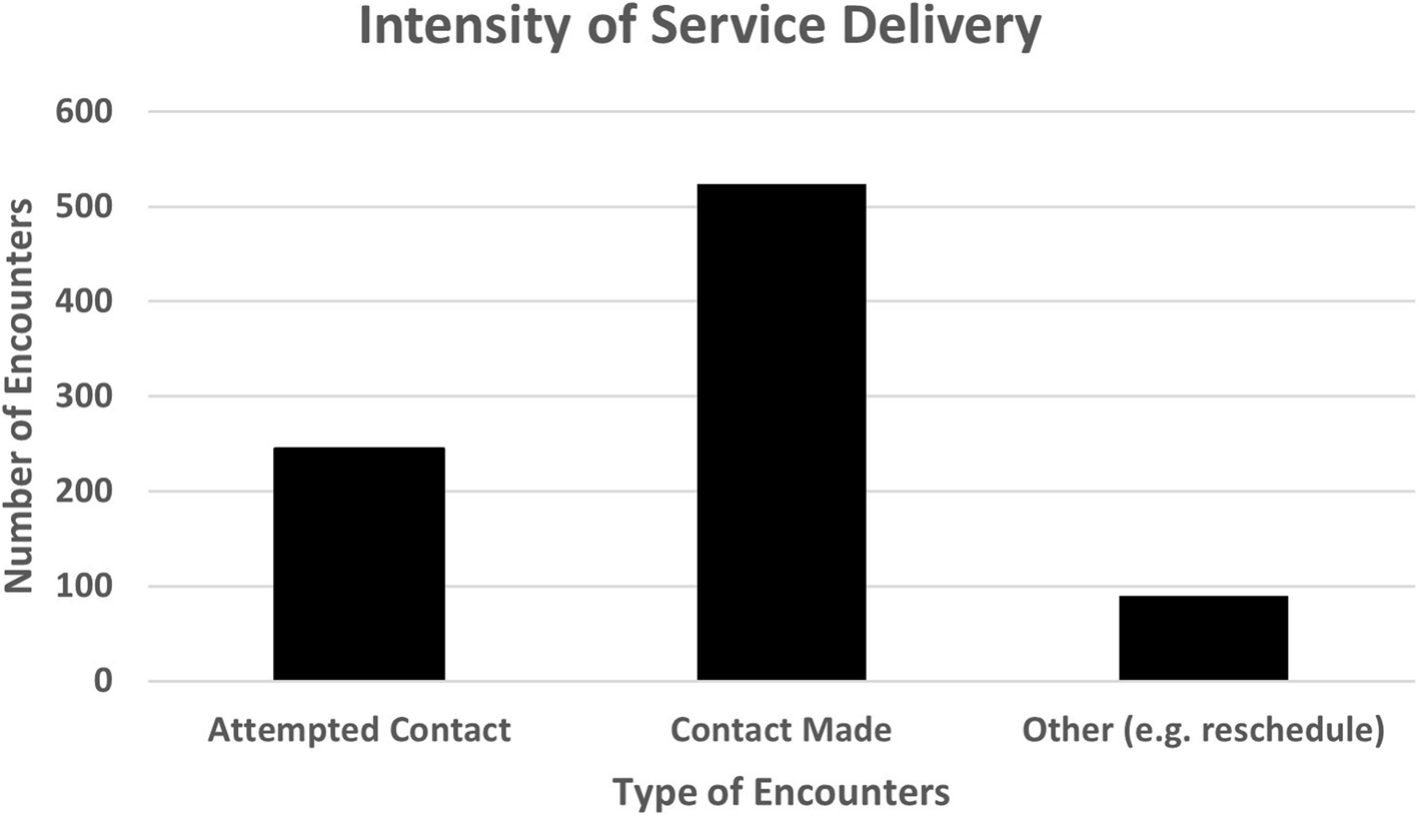
Intensity of service delivery

Over the course of follow-up, individuals were frequently engaged with the CHANGE team (Fig. 2). For the 86 participants enrolled to date, there were over 250 attempted contact encounters, over 500 encounters where contact was made, and nearly 100 other encounters including rescheduling. Encounter form data revealed that over half (52%) of the encounters resulted in direct services provided, including between attempted contacts to the participant, calls to schedule appointments, and providing services such as assistance with homeless verification and housing applications, HIV counseling and testing, and advocacy. Services were provided 1 to 61 times per individual during study enrollment, and a mean of 28% of contacts involved service provision. Over 92% of encounters were provided by Liberty housing and vocational case managers.

## Discussion

In Project CHANGE, we are engaging a highly vulnerable population of people experiencing CJS involvement, homelessness, and comorbid medical and psychiatric disorders. We found high rates of literal homelessness and unstable housing among people who are involved in the CJS, including through community supervision. Despite high unmet need, we have been able to successfully retain participants and deliver services at high intensity and frequency.

We provide a holistic approach to address needs through comprehensive integrated services, using a person-centered “one-stop shop” model, where participants have access to behavioral healthcare, housing and vocational case management, and medical services in a single location. The approach deployed here diverges from several well-established housing models. A more traditional “treatment first” approach to housing requires that people first stabilize with intensive community-based treatment for serious mental illness prior to being housed, thereby setting an often insurmountable barrier to housing. In contrast, “housing first” is often paired with assertive community treatment or case management [41]. In a large multisite study in Canada with 5 concurrent randomized controlled trials (known as *At Home/Chez Soi*), people with serious mental illness who were randomized to “housing first,” as compared to “treatment first,” experienced more rapid housing stability and improved psychological quality of life [42]. There is less evidence that “housing first” improves health status or severity of psychiatric symptoms, promotes recovery from substance use, or increases social capital or employment [41, 42, 45, 46]. In contrast to “housing first” and to meet gaps in existing services, we used a fully integrated model in which baseline screening for medical, psychiatric, housing, vocational, and substance use treatment needs was followed by on-site delivery of services to meet all identified needs in a “one-stop shop.”

Project CHANGE was initially designed with the hypothesis that enabled engagement in integrated comprehensive services would be stabilizing, and secondarily result in less CJS involvement. This premise is based on observations that homelessness is associated with rearrest for low-risk offenses [47], and supported by findings from prior pilot projects. For example, the “Returning Home-Ohio” study of 244 people with disabilities returning to communities from prison found that intervention group participants were significantly less likely than control group participants to be rearrested or reincarcerated, though arrest rates were low in both groups [16]. In the *At Home/Chez Soi* trials, participants receiving “housing first” across sites also experienced less frequent arrests for new offenses, though impact of housing interventions on CJS involvement have not been replicated in other randomized and non-randomized studies of housing interventions for people with psychiatric disorders in Canada [43, 44]. Elsewhere, other trials of housing interventions have similarly not observed an effect of housing intervention on CJS involvement, defined as number of jail stays [48] and re-incarceration [49]. These findings may reflect increased monitoring of people who are homeless and under community supervision, or varied definitions of CJS involvement. Impact of the CHANGE intervention on CJS involvement (broadly defined) will be reported once data collection is complete.

For people involved in the CJS, navigation of services can be particularly challenging for individuals experiencing co-occurring medical, psychiatric, and substance use issues—a complex problem that calls for integrated services. Full service integration may not be possible everywhere but, even when services are not fully integrated, co-location of services can be useful. A policy analysis found that integration of services enables clinicians and policymakers to treat patients (in this case, people who inject drugs) as a whole rather than in fragmented parts [50]. Similarly, in a randomly sampled study of 296 people who inject drugs in Ukraine, individuals who engaged in integrated or co-located healthcare had significantly higher quality healthcare indicator scores than individuals receiving siloed care and were more likely to engage in care than their non-co-located counterparts [51]. We found that CHANGE participants had many needs to address. Through a one-stop-shop model, participants have access to case management, medical care, and behavioral health, allowing them to care for their needs by addressing them in totality. These findings echo previous calls for the expansion of integrated service delivery that is patient-centered and low barrier to entry.

While delivering integrated housing and care services for people involved in the CJS, it was especially critical to maintain regular contact to enable program retention. This required frequent outreach by study staff and a high intensity and frequency of contacts with service providers, which reflects the high need in this population. Engagement is time-, staffing- and resource-intensive, requiring sufficient funding to provide wraparound support, which potentially threatens sustainability.

We encountered some additional challenges delivering intensive services during the COVID-19 pandemic, as have many community-based service programs. Under the March 31, 2020 executive order of Connecticut’s Governor, people staying in homeless shelters or visiting warming centers were relocated to alternative housing (known as “COVID hotels”) in an attempt to de-densify congregate settings to prevent disease spread [52]. While this provided an important temporary relief for literal homelessness, it meant that more people shifted to transitional housing while permanent housing options became unavailable [52]. People who are transitionally or temporarily housed may have fewer available housing options because they do not meet more traditional criteria for chronic homelessness and are thus not eligible for rapid re-housing. Individuals with income were unable to obtain affordable housing because eviction moratoria (which prohibits a tenant from being evicted due to not paying one’s rent) meant that there was slower turnover in housing and thus fewer options available [53, 54]. The pandemic temporarily limited economic opportunity and increased unemployment, which further restricted people’s ability to secure housing. Landlords began requiring that tenants earn an income three times the cost of rent, which was impossible for many of our participants. At the same time, the court system essentially ground to a halt, resulting in fewer arrests and convictions during the earliest phases of the pandemic in New England. While less CJS involvement was undoubtedly beneficial for people’s individual health and public health (where prisons and jails were disproportionately impacted by COVID-19), it also meant that fewer people were potentially eligible for participation in CHANGE. Because of University restrictions, all in person and enrollment clinical research activities were on hold for nearly one year, which significantly reduced the number of participants we could recruit, enroll, and interview. When we were unable to conduct in-person research we worked to find creative new ways to engage with participants, including conducting interviews virtually and providing electronic payments instead of traditional gift cards for remuneration.

This study has several limitations. It takes place in New England where there is Medicaid expansion, access to harm reduction programs, and an integrated system of jails and prisons, potentially limiting generalizability to other settings where these resources and coordination are not available. Second, much of our data are self-reported, potentially reflecting social desirability reporting biases, though CJS involvement was verified whenever possible. Third, many of our participants were recruited through our onsite syringe services program, potentially generating a sampling and selection bias because this was a sample of participants with high rates of opioid use disorder and injecting who were already engaging with harm reduction services. They may have differed in the severity and types of substance use disorders (more severe opioid use disorder) and service engagement (potentially more stable) than other people experiencing homelessness and CJS-involvement.

These limitations and challenges notwithstanding, Project CHANGE is a useful example of how comprehensive integrated care can address housing, behavioral health, and addiction treatment services for highly vulnerable populations at-risk for or living with HIV, by virtue of their involvement in the CJS.

## Conclusions

Prior community-based programs have focused on reducing housing instability in formerly incarcerated individuals, but there has been limited attention to improving physical and mental health outcomes, promoting recovery from substance use, increasing social capital, and promoting employment. Project CHANGE provides an example on how to holistically address medical and social needs of people who are CJS-involved, in a way that is person-centered and low barrier to entry.

## Supplementary Information

**Additional file 1: Supplemental Figure. **Behavioral Health Model for Vulnerable Populations (Gelberg et al.) as Theoretical Underpinning for Project CHANGE. Legend: CJ=criminal justice; CM=case management. 

## Data Availability

The datasets used and/or analyzed during the current study are available from the corresponding author on reasonable request.

## References

[1] Harding DJ, Wyse JJ, Dobson C, Morenoff JD (2014). Making ends meet after prison. J Policy Anal Manage.

[2] McKernan P. Homelessness and prisoner reentry: examining barriers to housing stability. Evidence based strategies that promote improved outcomes. Volunteers of America 2017. Available at: https://www.voa.org/homelessness-and-prisoner-reentry#Pat. Accessed 7 Sept 2022.

[3] Herbert CW, Morenoff JD, Harding DJ (2015). Homelessness and housing insecurity among former prisoners rsf the Russell Sage foundation. J Soc Sci.

[4] Harding DJ, Morenoff JD, Herbert CW (2013). Home is hard to find: neighborhoods, institutions, and the residential trajectories of returning prisoners. Ann Am Acad.

[5] Keene DE, Smoyer AB, Blankenship KM (2018). Stigma, housing and identity after prison. Sociol Rev.

[6] Aidala A, McAllister W, Yomogida M, Shubert V. Frequent Users Service Enhancement ‘FUSE’ Initiative: New York City Fuse II evaluation report. New York: Columbia University Mailman School of Public Health; 2003. Available at: https://shnny.org/uploads/CSH-FUSE-Evaluation.pdf. Accessed 7 Sept 2022.

[7] Russ E, Puglisi L, Eber G, Morse DS, Taxman F, Dupuis M, Ashkin E, Ferguson W (2021). Prison and jail reentry and health. Health Aff Health Pol Brief..

[8] Roman C, Travis J (2006). Where will I sleep tomorrow? Housing, homelessness, and the returning prisoner. Hous Policy Debate.

[9] Maruschak L, Bronson J. HIV in Prisons 2015- Statistical Tables. Bur Justice Stat. 2017;NCJ250641.

[10] Spaulding A, Seals R, Page M, Brzozowski A, Rhodes W, Hammett T (2009). HIV/AIDS among inmates of and releasees from US correctional facilities, 2006: declining share of epidemic but persistent public health opportunity. PLoS ONE.

[11] Rutledge R, Madden L, Ogbuagu O, Meyer JP (2018). HIV Risk perception and eligibility for pre-exposure prophylaxis in women involved in the criminal justice system. AIDS Care.

[12] Loeliger KB, Meyer JP, Desai MM, Ciarleglio MM, Gallagher C, Altice FL (2018). Retention in HIV care during the three years following release from incarceration: a cohort study. Annals Int Med (under review).

[13] Chen NE, Meyer JP, Avery AK, Draine J, Flanigan TP, Lincoln T, Spaulding AC, Springer SA, Altice FL (2013). Adherence to HIV treatment and care among previously homeless jail detainees. AIDS Behav.

[14] Binswanger IA, Blatchford PJ, Mueller SR, Stern MF (2013). Mortality after prison release: opioid overdose and other causes of death, risk factors, and time trends from 1999 to 2009. Ann Intern Med.

[15] Lutze FE, Rosky JW, Hamilton ZK (2014). Homelessness and Reentry: A multisite outcome evaluation of Washington State's reentry housing program for high risk offenders. Crim Justice Behav.

[16] Fontaine J, Gilchrist-Scott D, Roman J, Taxy S, Roman C. Supportive housing for returning prisoners: outcomes and impacts of the returning home - Ohio pilot project. The Urban Institute; 2012. p. 1–62.

[17] National Institute of Justice. Recidivism. Available at: https://nij.ojp.gov/topics/corrections/recidivism. Accessed 7 Sept 2022.

[18] Bonfine N, Wilson AB, Munetz MR (2020). Meeting the needs of justice-involved people with serious mental illness within community behavioral health systems. Psychiatr Serv.

[19] Gardner TM, Samuels PN, Nikolic S, Woodworth AM, Fleshler D. Health and Justice: bridging the gap. Lessons from New York State initiatives to provide access to care after incarceration. Legal Action Center; 2018.

[20] Augustine D, Kushel M (2022). Community supervision, housing insecurity, & homelessness. Ann Am Acad Pol Soc Sci.

[21] Gelberg L, Andersen R, Leake B (2000). The Behavioral Model for Vulnerable Populations: application to medical care use and outcomes for homeless people. Health Serv Res.

[22] WelfareInfo. Poverty Rate in New Haven, Connecticut. 2017. Available at: https://www.welfareinfo.org/poverty-rate/connecticut/new-haven/. Accessed 7 Sept 2022.

[23] Connecticut Department of Public Health. Connecticut department of public health drug overdose monthly report. 2021. Available at: https://portal.ct.gov/-/media/DPH/Injury-Prevention/Opioid-Overdose-Data/Monthly-Death-Reports/December-2021_2020-and-2019-Drug-Overdose-Deaths-Monthly-Report_Connecticut_1-20-2022_Final.pdf. Accessed 7 Sept 2022.

[24] Yale School of Medicine, Yale Clinical and Community Research. Community Health Care Van. Available at: https://medicine.yale.edu/intmed/infdis/yccr/chcv/. Accessed 7 Sept 2022.

[25] Liberty Community Services. Programs. Available at: https://www.libertycs.org/programs. Accessed 7 Sept 2022.

[26] National Human Services Data Consortium, Department of Housing and Urban Development. Homelessness 101. Available at: https://files.hudexchange.info/course-content/homelessness-101/Homelessness-101-Slides.pdf. Accessed 4 May 2020.

[27] Substance Abuse and Mental Health Services Administration (SAMHSA). Screening, Brief Intervention, and Referral to Treatment (SBIRT). Available at: https://www.samhsa.gov/sbirt/about. Accessed 7 Sept 2022.

[28] Harris P, Taylor R, Minor B, Elliott V, Fernandez M, O'Neal L, McLeod L, Delacqua G, Delacqua F, Kirby J (2019). The REDCap consortium: Building an international community of software platform partners. J Biomed Inform.

[29] Harris P, Taylor R, Thielke R, Payne J, Gonzalez N, Conde J (2009). Research electronic data capture (REDCap)—A metadata-driven methodology and workflow process for providing translational research informatics support. J Biomed Inform.

[30] Moore S, Sharp A. Addiction Severity Index (ASI): Using the ASI Assessment. Available at: https://americanaddictioncenters.org/rehab-guide/asi-addiction-severity-index-assessment. Accessed 9 Sept 2022.

[31] Grahn R, Padyab M (2020). The predictability of the Addiction Severity Index criminal justice assessment instrument and future imprisonment: A Swedish registry study with a national sample of adults with risky substance use. Drug Alcohol Depend.

[32] Meyer JP, Price CR, Ye Y, Qin Y, Tracey D, Demidont AC, Melbourne K, Altice FL (2022). A PrEP demonstration project using ehealth and community outreach to justice-involved cisgender women and their risk networks. AIDS Behav.

[33] RAND Corporation. 12-Item Short Form Survey (SF-12). Available at: https://www.rand.org/health-care/surveys_tools/mos/12-item-short-form.html. Accessed 7 Sept 2022.

[34] Soh S-E, Morello R, Ayton D, Ahern S, Scarborough R, Zammit C, Brand M, Stirling RG, Zalcberg J (2021). Measurement properties of the 12-item Short Form Health Survey version 2 in Australians with lung cancer: a Rasch analysis. Health Qual Life Outcomes.

[35] Ware J, Kosinski M, Keller SD (1996). A 12-Item Short-Form Health Survey: construction of scales and preliminary tests of reliability and validity. Med Care.

[36] Kroenke K, Spitzer RL, Williams JB (2001). The PHQ-9: validity of a brief depression severity measure. J Gen Intern Med..

[37] Radloff LS (1977). A self report depression scale for research in the general populations. Appl Psychol Measure.

[38] National Institute on Drug Abuse. The NIDA Modified ASSIST, version 1.0. Available at: https://nida.nih.gov/sites/default/files/pdf/nmassist.pdf. Accessed 7 Sept 2022.

[39] Skinner HA (1982). The drug abuse screening test. Addict Behav.

[40] Yudko E, Lozhkina O, Fouts A (2007). A comprehensive review of the psychometric properties of the drug abuse screening test. J Subst Abuse Treat.

[41] Polcin DL (2016). Co-occurring substance abuse and mental health problems among homeless persons: suggestions for research and practice. J Soc Distress Homeless.

[42] Aubry T, Bourque J, Goering P, Crouse S, Veldhuizen S, LeBlanc S, Cherner R, Bourque P, Pakzad S, Bradshaw C (2019). A randomized controlled trial of the effectiveness of housing first in a small Canadian City. BMC Public Health.

[43] Somers JM, Rezansoff SN, Moniruzzaman A, Palepu A, Patterson M (2013). Housing first reduces re-offending among formerly homeless adults with mental disorders: results of a randomized controlled trial. PLoS ONE.

[44] Leclair MC, Deveaux F, Roy L, Goulet MH, Latimer EA, Crocker AG (2019). The impact of housing first on criminal justice outcomes among homeless people with mental illness: a systematic review. Can J Psychiatry.

[45] Manhattan Institute of Policy Research, Eide S. Housing First and Homelessness: The Rhetoric and the Reality. 2020. Available at: https://www.manhattan-institute.org/housing-first-effectiveness. Accessed 7 Sept 2022.

[46] Pakzad S, Bourque P-É, Bourque J, Aubry T, Gallant L, LeBlanc SR, Tivendell J (2017). A comparison of the use of physical and mental health services by homeless people with severe mental health problems in the moncton area through the at home/Chez Soi program. Can J Commun Ment Health.

[47] Jacobs LA, Gottlieb A (2020). The effect of housing circumstances on recidivism: evidence from a sample of peopleon probation in San Francisco. Crim Justice Behav..

[48] Raven MC, Niedzwiecki MJ, Kushel M (2020). A randomized trial of permanent supportive housing for chronically homeless persons with high use of publicly funded services. Health Serv Res.

[49] Luong L, Lachaud J, Kouyoumdjian FG, Hwang SW, Mejia-Lancheros C (2021). The impact of a housing first intervention and health-related risk factors on incarceration among people with experiences of homelessness and mental illness in Canada. Can J Public Health.

[50] Sylla L, Bruce R, Kamarulzaman A, Altice F (2007). Integration and co-location of HIV/AIDS, tuberculosis and drug treatment services. Int J Drug Policy.

[51] Bachireddy C, Soule MC, Izenberg JM, Dvoryak S, Dumchev K, Altice FL (2014). Integration of health services improves multiple healthcare outcomes among HIV-infected people who inject drugs in Ukraine. Drug Alcohol Depend.

[52] State of Connecticut, Department of Housing. Notice and order regarding executive order 7P safe housing for people experiencing homelessness. 2020. Available at: https://portal.ct.gov/-/media/DOH/Notice--Order-for-homeless-shelters.pdf. Accessed 7 Sept 2022.

[53] National Low Income Housing Coalition. The Gap: A shortage of affordable rental homes. 2021. Available at: https://nlihc.org/gap. Accessed 7 Sept 2022.

[54] Housing Solutions. What the eviction moratorium means for landlords. Available at: https://www.housingsolutionstulsa.org/what-the-eviction-moratorium-means-for-landlords/#:~:text=The%20eviction%20moratorium%20prohibits%20any,for%20non%2Dpayment%20of%20rent. Accessed 7 Sept 2022.

