# Supplementary material for: Experience delivering an integrated service model to people with criminal justice system involvement and housing insecurity
Source: BMC Public Health. 2023 Feb 2;23:222. doi: 10.1186/s12889-023-15108-w (PMC9892679; doi:10.1186/s12889-023-15108-w)
Supplement: Supplementary file 1 — Additional file 1: Supplemental Figure. Behavioral Health Model for Vulnerable Populations (Gelberg et al.) as Theoretical Underpinning for Project CHANGE. Legend: CJ=criminal justice; CM=case management. [file 12889_2023_15108_MOESM1_ESM.pptx]

## Slide 1
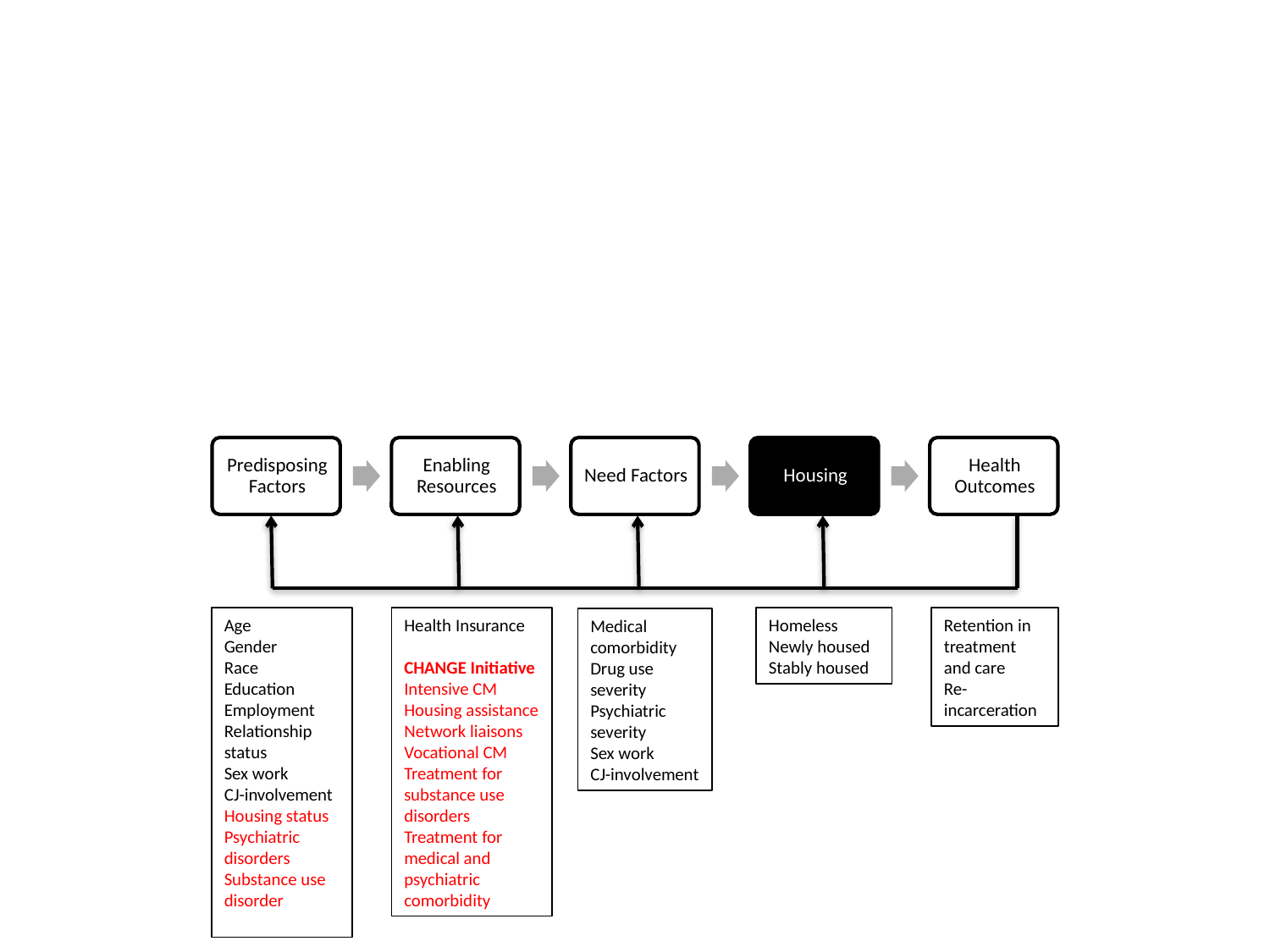

Age
Gender
Race
Education
Employment
Relationship status
Sex work
CJ-involvement
Housing status
Psychiatric disorders
Substance use disorder
Health Insurance
CHANGE Initiative
Intensive CM
Housing assistance
Network liaisons
Vocational CM
Treatment for substance use disorders
Treatment for medical and psychiatric comorbidity
Homeless
Newly housed
Stably housed
Retention in treatment and care
Re-incarceration
Medical comorbidity
Drug use severity
Psychiatric severity
Sex work
CJ-involvement
